# Radial and median nerves distal peripheral tension after reverse shoulder arthroplasty: a cadaveric study

**DOI:** 10.1016/j.jseint.2024.03.013

**Published:** 2024-04-16

**Authors:** Gregory Cunningham, Lauryne Bernardo, Rodrigo Brandariz, Nicolas Holzer, Daniel Da Rocha, Jean-Yves Beaulieu

**Affiliations:** a Shoulder and Elbow Center La Colline, Geneva, Switzerland; b Division of Orthopaedics and Trauma Surgery, Department of Surgery, Geneva University Hospitals, Geneva, Switzerland; c Faculty of Medicine, University of Geneva, Geneva, Switzerland

**Keywords:** Reverse shoulder arthroplasty, Peripheral nerve injury, Upper limb positioning, Distal nerve tensioning, Distal peripheral nerve, Neuropathy, Reverse shoulder arhtroplasty, Nerve tension, Cadaveric study, Tensiometer, Radial nerve, Median nerve

## Abstract

**Background:**

Peripheral nerve injury is a recognized complication after reverse shoulder arthroplasty (RSA) that has mainly been studied at the level of the brachial plexus and its proximal branches. However, the impact of RSA on distal peripheral nerves and the influence of elbow and wrist position is not known. This cadaveric study aimed to analyze the effect of RSA implantation and upper limb position on tension in the distal median and radial nerves. The hypothesis was that RSA increased distal nerve tension, which could be further affected by elbow and wrist position.

**Methods:**

12 upper limbs in 9 full fresh-frozen cadavers were dissected. Nerve tension was measured in the median nerve at the level of the proximal arm, elbow, and distal forearm, and in the radial nerve at the level of the elbow, using a customized three-point tensiometer. Measurements were carried out before and after RSA implantation, using a semi-inlay implant (Medacta, Castel San Pietro, Switzerland). Two different configurations were tested, using the smallest and largest available implant sizes. Three upper-limb key positions were considered (plexus at risk, plexus relief, and neutral), from which the effect of elbow and wrist position was further tested.

**Results:**

RSA implantation significantly increased median and radial nerve tension throughout the upper limb. The distal nerve segments were particularly dependent on elbow and wrist position. The plexus at risk position induced the most tension in all nerve segments, especially with the large implant configuration. On the other hand, the plexus relief position induced the least amount of tension. Flexing the elbow was the most efficient way to decrease nerve tension in all tested nerve segments and key positions. Wrist flexion significantly decreased nerve tension in the median nerve, whereas wrist extension decreased tension in the radial nerve.

**Conclusion:**

RSA significantly increases tension in the median and radial nerves and makes them more susceptible to wrist and elbow positioning. The mechanism behind distal peripheral neuropathy after RSA may thus result from increased compression of tensioned nerves against anatomical fulcrums rather than nerve elongation alone. Elbow flexion was the most effective way to decrease nerve tension, while elbow extension should be avoided when implanting the humeral component. Further studies are needed to assess the ulnar nerve.

Reverse shoulder arthroplasty (RSA) has been used to treat an increasingly wide range of conditions.^10^^,^^14^

Even though clinical results and long-term survivorship are still improving with evolving knowledge, newer implant designs, and surgical techniques, the reported rate of complication remains substantial and highly variable, ranging up to 15%-24%.^8^ According to Zumstein et al’s systematic review, the most frequent complication is instability (6.9%), and the least frequent neurologic lesions (1.7%).^25^ However, neurologic lesions seem to have been underreported, with recent retrospective clinical studies reporting rates as high as 19% to 22% following RSA.^8^ An electromyographic study by Lädermann et al found subclinical neurologic lesions in almost 50% of patients undergoing RSA compared to 4.3% for anatomic shoulder arthroplasty.^1^

Neurologic lesions after RSA are reported with varying incidences of nerve involvement and mainly present as mixed plexopathies.^1^^,^^12^^,^^17^^,^^20^, ^21^, ^22^ The reported mechanisms of neurologic lesions after RSA are arm-lengthening, retractor positioning, arm position during the procedure, and postoperative immobilization.^13^^,^^15^^,^^22^ Other patient-related risk factors are severe preoperative stiffness (external rotation <10°), female sex, and previous surgeries.^1^^,^^17^^,^^18^

Several clinical and cadaveric studies have shown that RSA induces significant elongation and an increase in tension at the level of the brachial plexus and its proximal branches.^15^^,^^16^^,^^20^ It is however not known how the increase in nerve tension caused by RSA affects distal peripheral nerves.

Few clinical studies have specifically examined the incidence of postoperative distal peripheral neuropathy (DPN) following RSA. In a retrospective clinical study, Thomasson et al^23^ found a rate of 12.3% DPN after RSA, compared to 7.1% after anatomic shoulder arthroplasty and 2.7% after rotator cuff repair. DPN mainly consisted of cubital followed carpal tunnel syndromes, requiring further surgical decompression in 14.3% of the cases. They stated that they were not able to explain the underlying mechanism of DPN following RSA.

The purpose of this cadaveric study was thus to evaluate the influence of RSA implantation as well as upper-limb position on distal peripheral nerve tension. It was hypothesized that RSA would lead to a significant increase in distal peripheral nerve tension throughout the arm, with further variation caused by elbow and wrist position.

## Material and methods

### Specimen preparation

**Figure 1:**
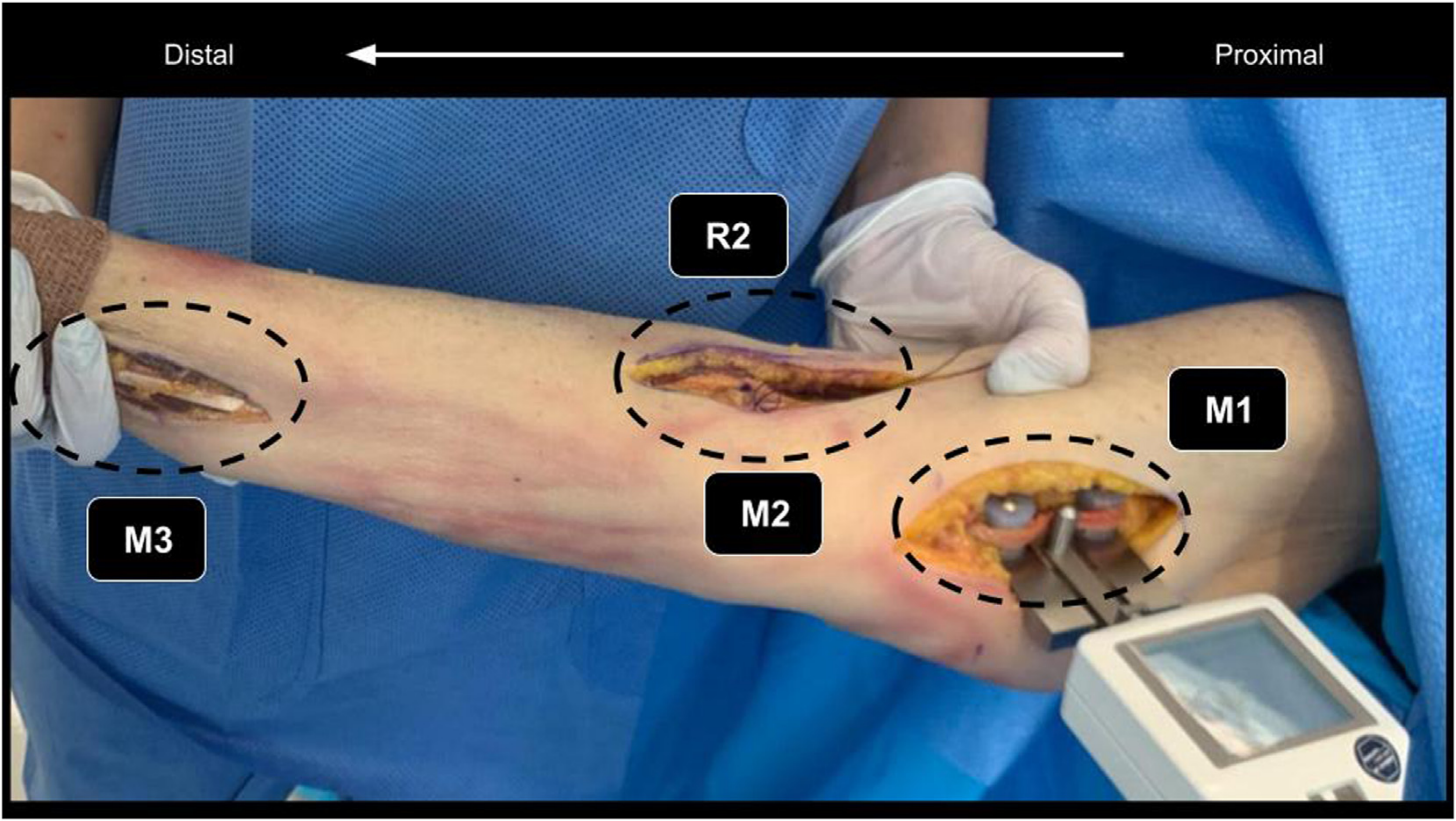
Approaches sites. Picture showing the three approached sites. From proximal to distal (*Right* to *Left*). M1: Median nerve in the Distal humerus. M2: Median nerve in the Antecubital Fossa. R2: Radial nerve in the Antecubital Fossa. M3: Median nerve in the Volar distal radius.

This cadaveric study involved 12 shoulders (8 right and 4 left) in 9 fresh-frozen entire cadavers (6 males, 3 females, aged 65-84 years). Three anatomic locations were approached to expose the peripheral nerves (Fig. 1): the distal humerus, 10 centimeters proximal from the medial epicondyle (M1), the antecubital fossa (M2), and the volar distal radius (M3). The dissections were carried out by 2 surgeons specialized in upper limb and peripheral nerve surgery (GC, JYB). The nerves were carefully exposed with maximal surrounding soft tissue preservation to allow the minimal amount of space required for a tensiometer.

### Reverse shoulder arthroplasty implantation and configuration

The cadavers were positioned in the beach chair position with the trunk flexed at 45°. A deltopectoral approach with subscapularis tenotomy was performed, and the arthroplasties were implanted following the surgical steps of the technical guide provided by the industry (Medacta, Castel San Pietro, Switzerland). A 27 mm pegged baseplate was implanted and fixed with two peripheral screws on the glenoid. A semi-inlay stem with a +0 metaphysis was implanted in the humerus. Two different prosthetic configurations were then tested, using the least and most lateralizing and distalizing configurations available at the time of the experiment. The first configuration (P1) consisted in a 36 mm glenosphere, +0mm polyethylene insert (PE) in the 145° neck-shaft angle position. The second (P2) consisted in a 42 mm glenosphere, +6 mm PE, and a 155° neck-shaft angle position.

### Peripheral nerve tension measurement

**Figure 2:**
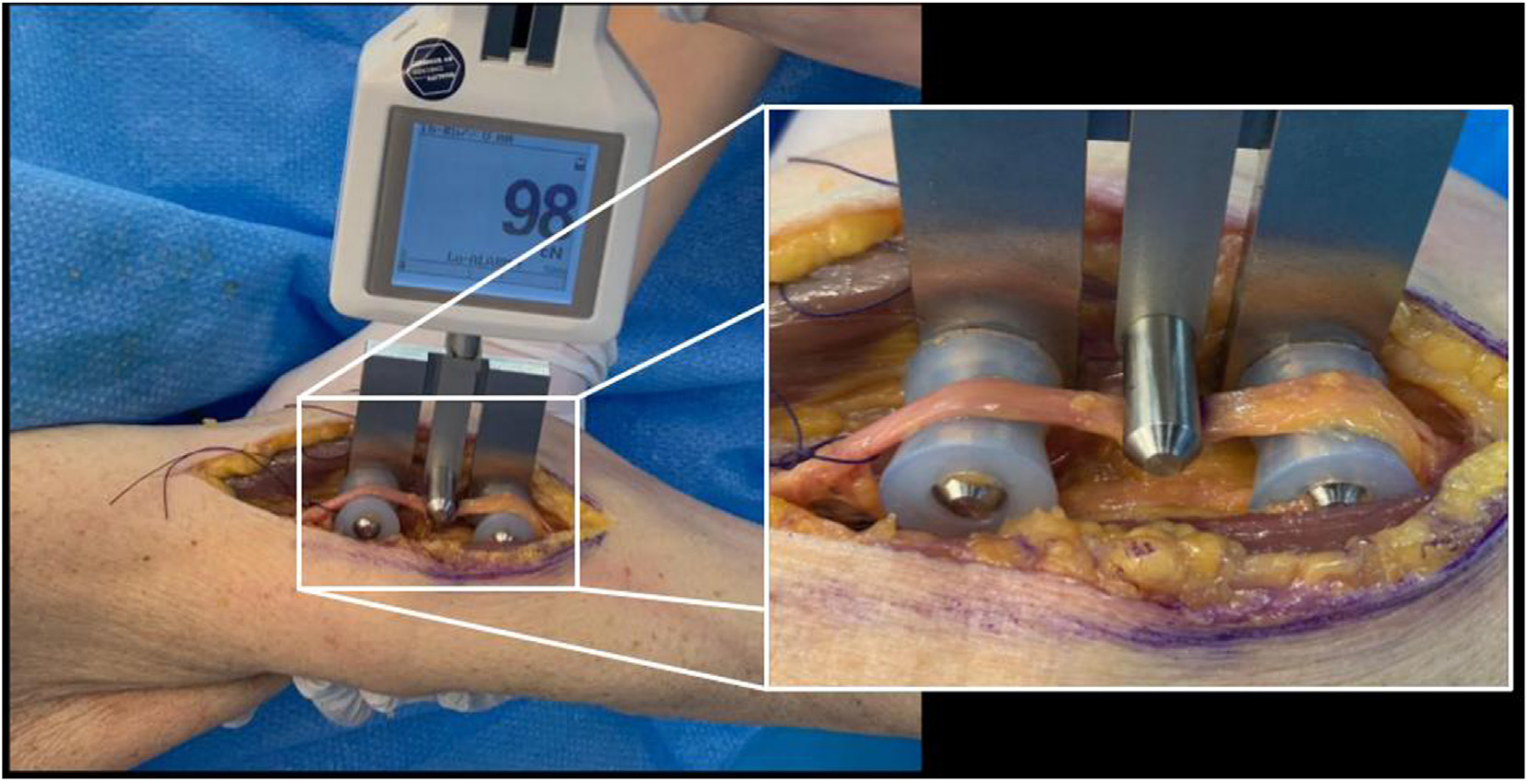
Measurement technique. Picture showing the measurement technique of the median nerve in the antecubital fossa using a 3-point customized electronic tensiometer.

A 3-point customized electronic tensiometer (Hans Schmidt & Co GMBH, Waldkraiburg, Germany) with 15mm diameter stainless-steel and 3D-printed plastic pins, and a tension range from 50 to 5000 cN, was utilized to measure nerve tension (Fig. 2). The measurements were performed by 3 observers (GC, LR, DDR) at a single time point to minimize repetitive nerve handling and subsequent risk of nerve creep. They were recorded once the continuously recorded value had stabilized for at least 10 seconds. Measurements were performed in the native joint state (N) and after the P1, and P2 RSA implantation. The nerves were marked with ink prior to the measures to accurately reposition the center of the tensiometer.

Tension in the median nerve was measured in the distal humerus (M1), antecubital fossa (M2), and distal volar radius (M3). Tension in the radial nerve was measured in the antecubital fossa (R2). The distal radial nerve and the ulnar nerve were not included in this study, as they were too thin or slack to obtain reliable measures with the utilized device.^12^

### Upper limb positions

**Figure 3:**
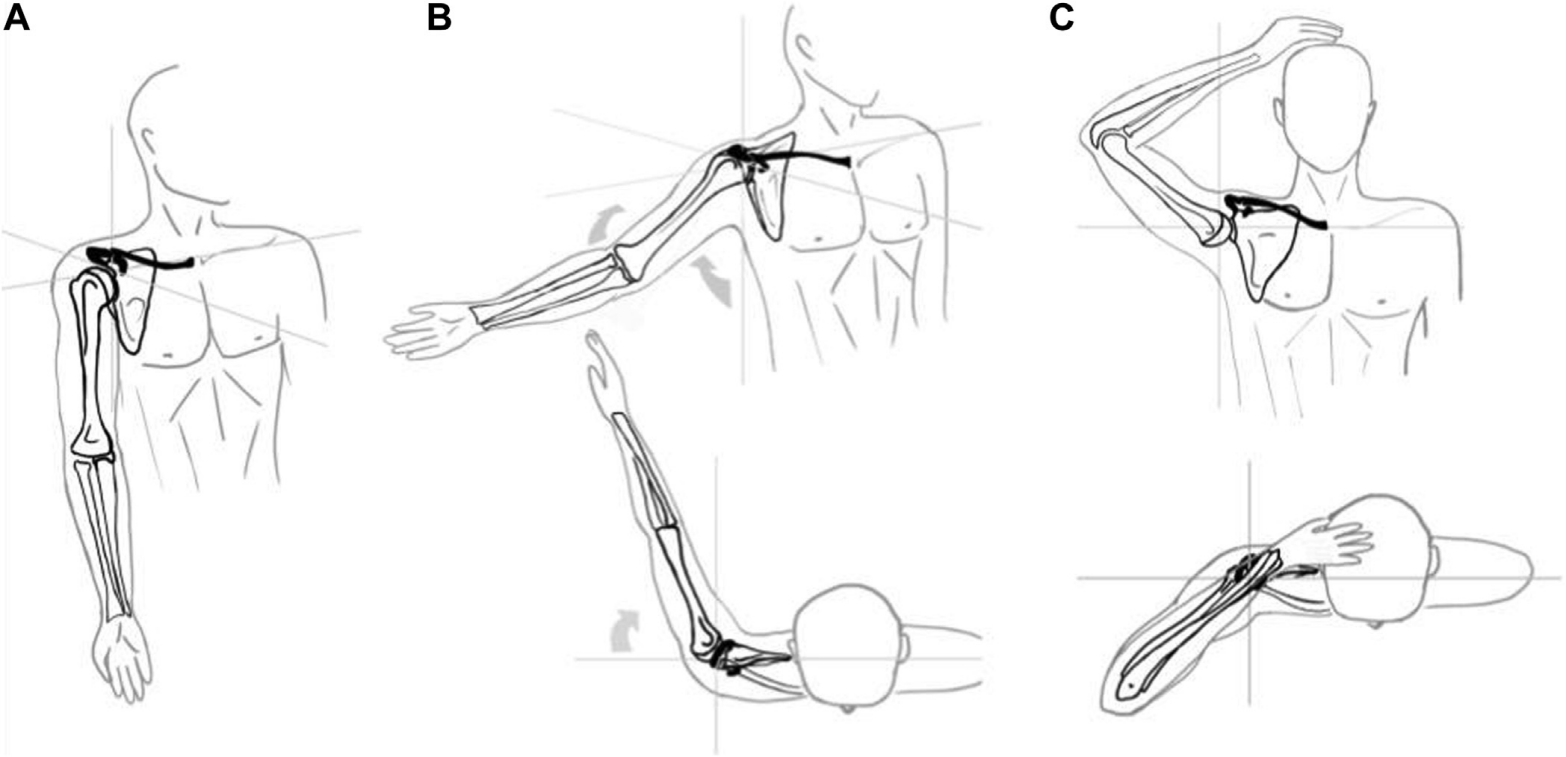
Baseline positions. Illustration of the three tested baseline positions. (**A**) Neutral, with the arm to the body. (**B**) Plexus at risk, with the arm in extension and external rotation. (**C**) Plexus relief, with the hand placed on the head and the shoulder in abduction and 45° flexion.

For each of the three settings (N, P1, and P2), the upper limb was positioned in three different baseline key configurations (Fig. 3): ‘Neutral’, with the arm to the body; ‘Plexus at risk’, with the arm in extension and external rotation (a position that has been found to have the maximum nervous tension in the study by Lenoir et al),^15^ ‘Plexus relief’, with the hand placed on the head and the shoulder in abduction and 45° flexion, as described in Bakody’s shoulder abduction test.^5^^,^^6^

**Figure 4:**
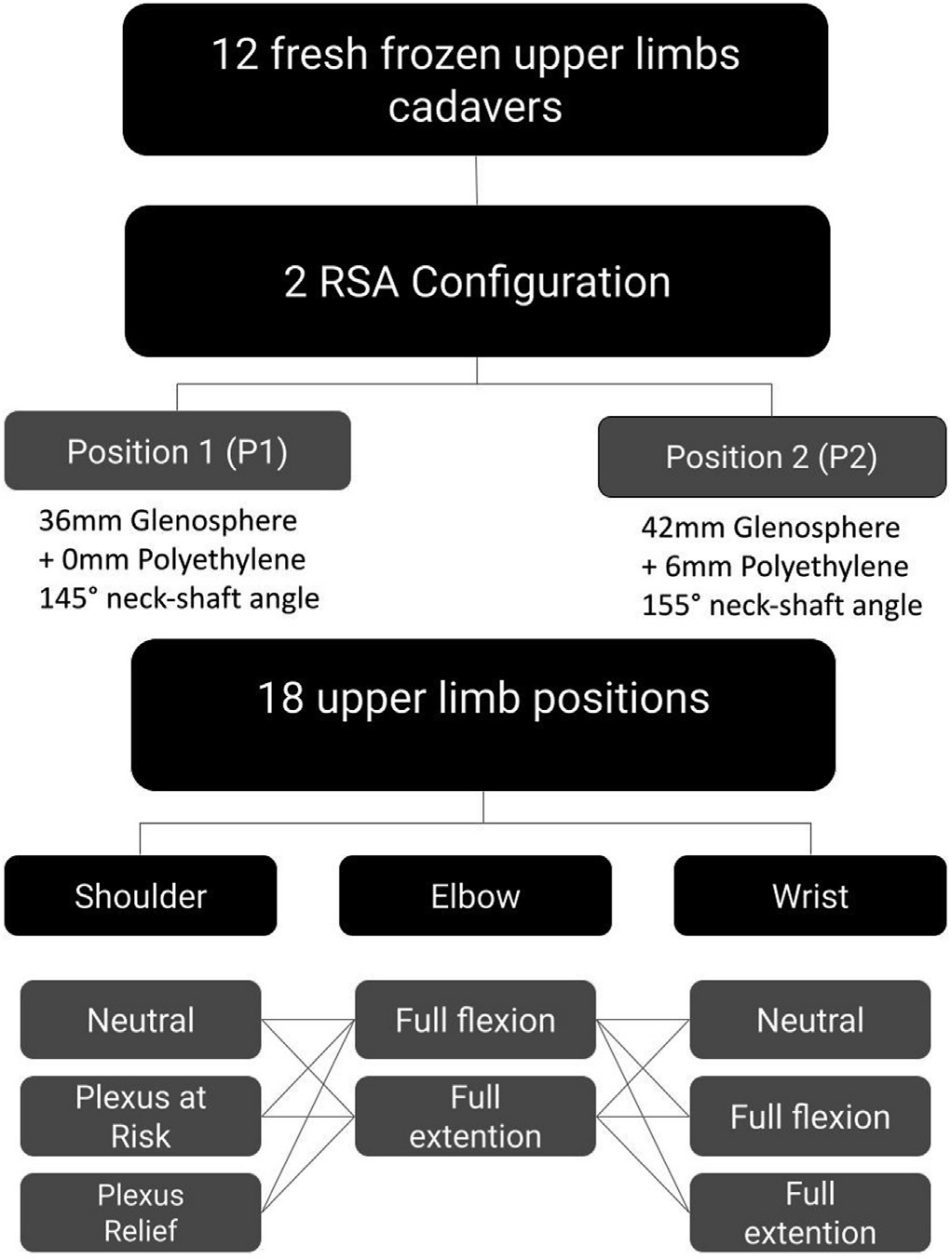
Specimen selection flowchart. Flowchart representing the specimen selection, tested joint configuration, locations where nerve tension was measured, and the different upper limb positions.

From all three baseline positions, variation of nerve tension was recorded depending on elbow and wrist flexion and extension, totaling 18 different key positions. (Fig. 4)

### Statistical analysis

Variables are reported as means or differences in cN and proportions (%). Paired T-test and Mann-Whitney U test were used to compare mean differences between groups for parametric and nonparametric data, respectively. A significant *P* value was set at .05. Statistical analysis was done with StatPlus (Version 8; AnalystSoft Inc., Alexandria, VA, USA).

## Results

### Overall influence of RSA on nerve tension

**Figure 5:**
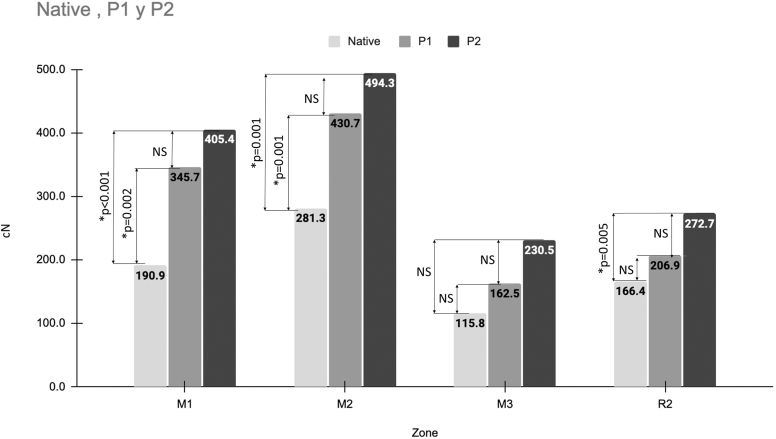
Overall tension in median and radial nerves. Bar Graph showing the overall tension (cN) in the three tested segments of the median nerve (M1, M2 and M3), and the radial nerve in the R2 segment. Nerve tension was measured in the native joint, and after RSA implantation with the smallest (P1) and largest (P2) implant configuration. *RSA*, reverse shoulder arthroplasty.

Overall, RSA significantly increased median nerve tension regardless of the joint configuration (P1 or P2) and the level of the arm (M1 and M2). There was however no significant increase at the wrist (M3). For the radial nerve (R2), there was only a significant difference between the native setting and the P2 RSA configuration. There was a slight but nonsignificant tension increase in all nerves and segments between P1 and P2 joint configurations (Fig. 5).

### Influence of baseline key upper limb position on nerve tension

**Figure 6:**
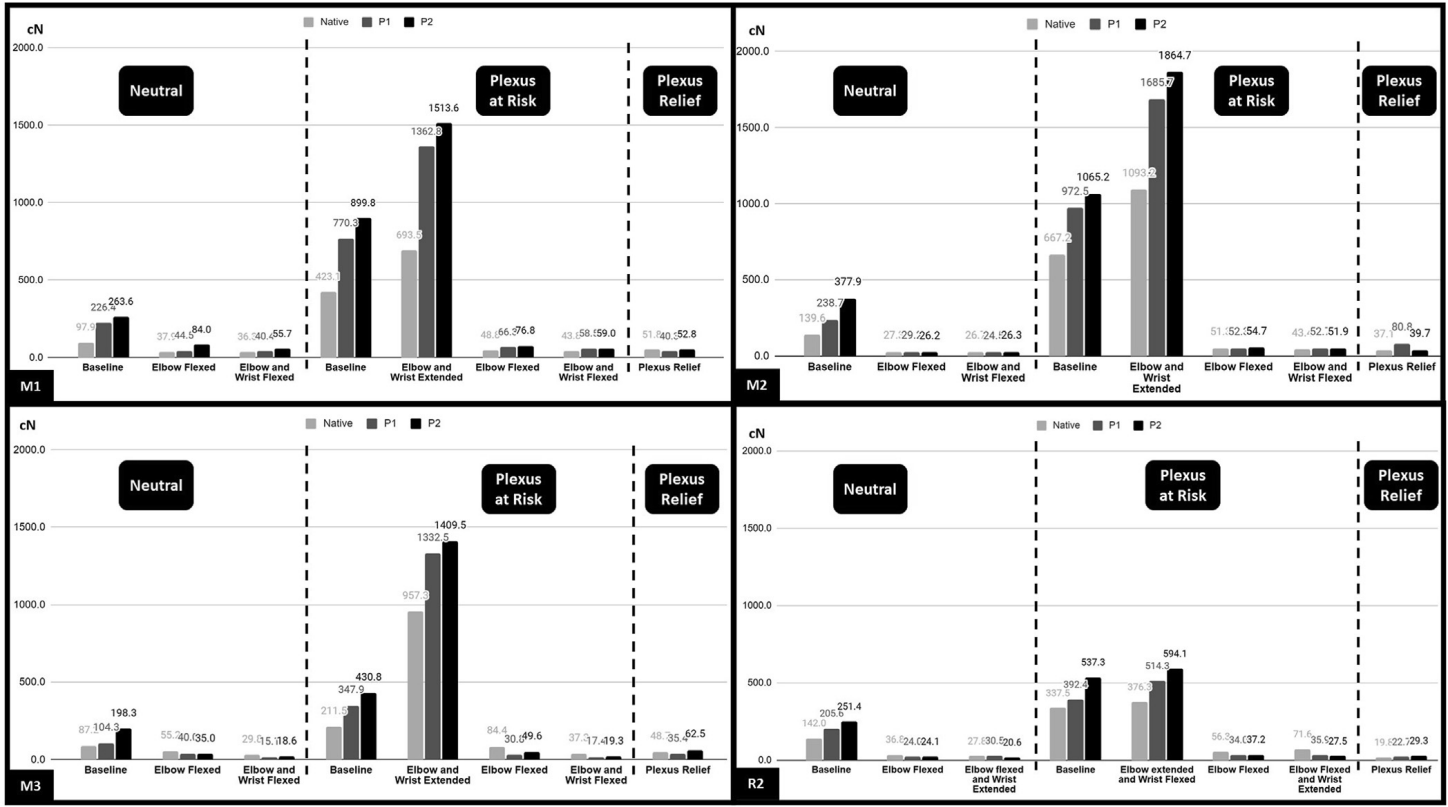
Influence of arm Position on median and radial nerve tension. Bar Graphs representing the tension variations of the median and radial nerves in the different tested anatomic segments (M1, 2 and 3; and R2), in three different global positions (Neutral, Plexus at risk, Plexus Relief).

The results of the following section are summarized in Fig. 6. For all joint configurations (native, P1 and P2), the ‘Plexus at risk’ position resulted in the highest nerve tension in all nerves and all segments, and the ‘Plexus relief’ the lowest. Compared to the ‘Neutral’ position, the ‘Plexus at risk’ significantly increased tension in all nerve segments (all *P* ≤ .001), apart from the M3 segment in the Native configuration where there was a slight nonsignificant increase. Compared to the Neutral position, the Plexus relief position significantly decreased nerve tension in all segments (all *P* < .001-0.5), except for M1 in the Native joint where the decrease was nonsignificant.

### Influence of elbow and wrist position in the neutral position

From the Neutral position, flexing the elbow induced a significant decrease in nerve tension in all analyzed nerve segments and joint configurations (all *P* values < .001-.5), except for M3 in the Native joint. The resulting nerve tension became similar to putting the arm in the ‘Plexus relief’ position. Further flexion of the wrist did not significantly decrease tension in any nerve segment and joint configuration.

### Influence of elbow and wrist position in the plexus at risk position

From the ‘Plexus at risk’ position, flexing the elbow substantially decreased nerve tension in all nerve segments and joint configurations (all *P* values < .001-.002), except for M3 in the Native joint. The resulting nerve tension was also similar to when the ‘Plexus relief’ position.

Wrist extension in the ‘Plexus at risk’ position significantly further increased nerve tension in all segments of the median nerve in all joint settings (all *P* values < .001), especially in the distal segment of the median nerve (M3) but didn’t significantly decrease tension in the radial nerve. Wrist extension in the ‘Plexus at risk’ was the tested configuration that induced the most nerve tension in the Median nerve, whereas wrist flexion in the ‘Plexus at risk’ position induced the most tension in the Radial nerve (all *P* values < .001).

## Discussion

**Figure 7:**
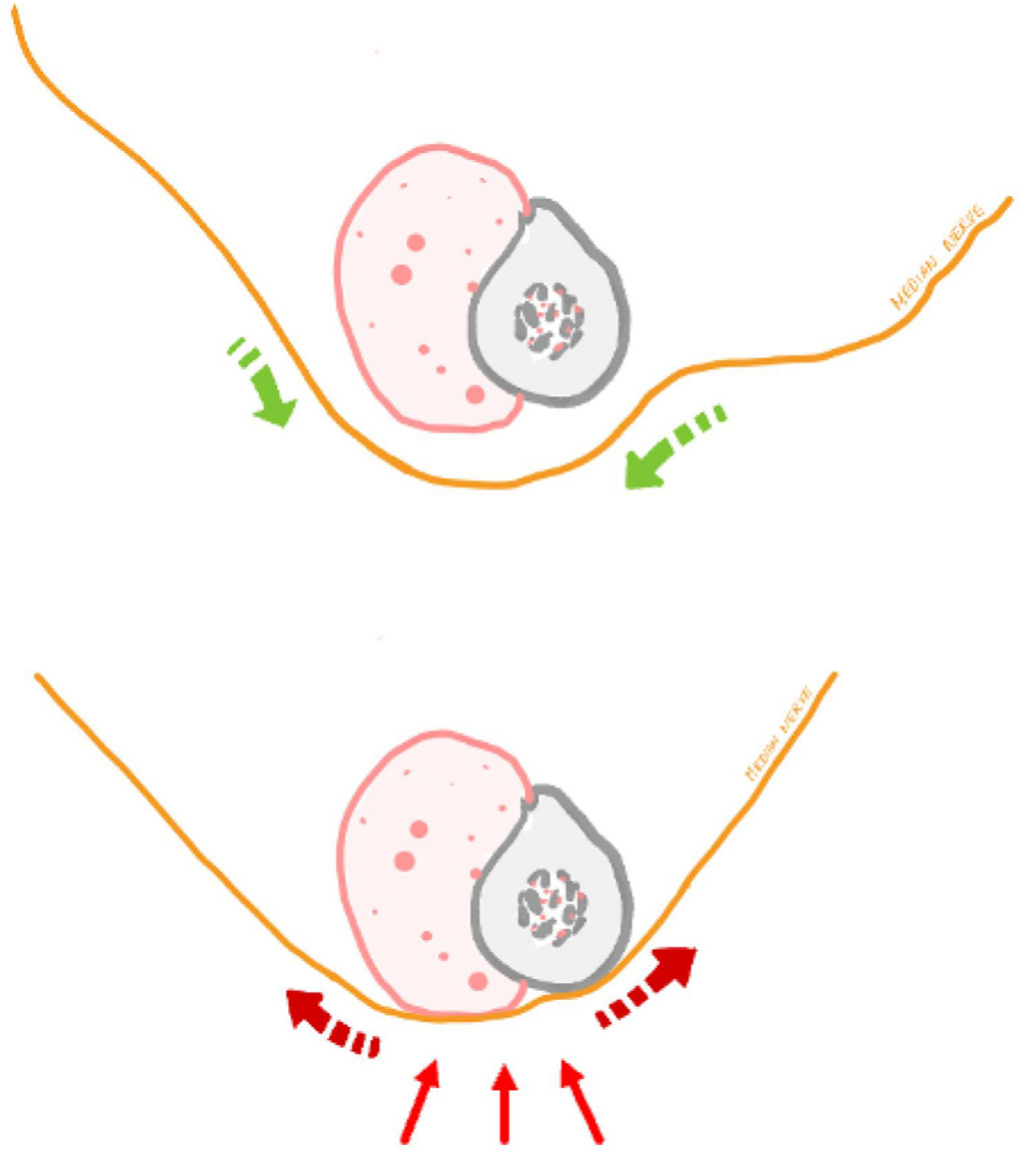
Fulcrum effect. Schematic illustration of the fulcrum effect of a tensioned nerve wrapping around an anatomically fixed pulley, increasing nerve compression against the anatomic structure.

RSA significantly increases tension in the median and radial nerves, making them more susceptible to wrist and elbow positioning. For the median nerve, tension is mostly increased at the level of the arm (M1) and at the elbow (M2) and slightly but not significantly at the level of the distal forearm (M3). This segment is mostly influenced by the position of the elbow and wrist, which was however amplified by RSA implantation. These key findings suggest that nerve tension may dissipate throughout the upper limb, following the physical laws of a cable and rigid pulley system, where the friction of the cable against the pulleys gradually dissipates tension.^7^ Therefore, DPN after RSA may be caused by an increased fulcrum effect exerted by adjacent anatomic structures compressing the tensioned nerves, previously hypothesized as an ‘extrinsic mechanism’. A substantial amount of symptomatic DPN after RSA could in fact be a decompensation of an underlying paucisymptomatic condition. In predominantly retrospective clinical studies on outcomes following shoulder arthroplasty, the preoperative condition of peripheral nerves is often overlooked and underreported, as attention and recorded clinical scores primarily focus on shoulder function. Neurologic lesions are therefore more prone to be reported as postoperative complications.^23^ A double crush syndrome, consisting in injury to the proximal plexus during surgery in addition to an underlying DPN could also be a causal factor. Nevertheless this theory remains debated and the first crush seems rather related to underlying systemic causes than dual nerve injury.^4^^,^^19^ Other factors may also play a role in postoperative DPN, such as inflammation, soft tissue swelling, hematoma, and prolonged immobilization. Nevertheless, in such cases, symptoms would be expected to be transient and resolve over the course of several weeks or months,^1^^,^^9^^,^^20^ as nerves have been found to adapt to elongation (creep) to a certain extent.^3^^,^^11^ However, a significant proportion of patients present ongoing DPN symptoms following RSA that require further surgical decompression.^23^ This could be explained by the long-lasting dynamic alterations caused by the aforementioned increased fulcrum and compressive pulley effect (Fig. 7).

This mechanism seems to be different from injuries to the proximal nerves around the shoulder, caused by arm lengthening, excessive traction on the arm during the procedure, or direct contact with retractors, largely examined in previous cadaveric and electrophysiologic studies.^15^^,^^16^^,^^18^^,^^20^ Marion et al^16^ measured nerve elongation rather than tension in the shoulder after RSA using 2 different Grammont-type implants in 20 cadaveric shoulders. They found significant elongation in all nerves except the radial nerve. This contrasts with our study, which also found a significant increase in radial nerve tension after RSA. Nerve elongation may thus not accurately reflect nerve tension, particularly in the radial nerve, which follows a relatively fixed course around the humerus. In an in vivo electromyographic study, Parisien et al^18^ found a nearly 5-fold increase in nerve alerts after RSA compared to anatomic total shoulder arthroplasty. Furthermore, most of the nerve alerts in both groups (65%) occurred while the arm was in external rotation. Therefore, peripheral nerves were not only affected by the type and design of the prosthesis but also by the position of the upper limb during the procedure. Using a similar methodology looking at 10 cadaveric shoulders after implanting a Grammont-type RSA, Lenoir et al^15^ also found that combined extension and external rotation, named “plexus at risk” position in this study, induced the most strain on the proximal nerve branches of the brachial plexus. Additionally, they found that incrementing the size of PEs only significantly increased tension in the axillary nerve (+3 to +9 mm), but not in the other tested nerves. However, all nerves showed significantly increased tension between tested implant sizes and the native joint, except for the ulnar nerve. We also found a nonsignificant increase in the median and radial nerve tension between the different tested implant configurations (P1 and P2), but significant increases in tension compared to the native joint. However, none of these studies have analyzed tension in distal peripheral nerves or the effect of elbow and wrist position.

In the present study, we also found that arm positioning played a critical role in distal peripheral nerve tension, and particularly the elbow and the wrist. The ‘Plexus at risk’ position induced the most tension in all nerve segments. Further extending and flexing the wrist in this position strongly increased nerve tension in the median nerve (especially the M3 segment) and radial nerve, respectively. Elbow flexion was however found to be the most effective way to reduce nerve tension in the ‘Plexus at risk’ and ‘Neutral’ position. It had a similar effect to putting the arm in the ‘Plexus relief’ position. This corroborates the findings of a cadaveric study by Bueno-Garcia et al who observed that elbow flexion alleviated strain in the median nerve at the level of the wrist caused by cervical contralateral flexion.^2^ These findings suggest that the use of commonly administered postoperative slings could be protective in patients with underlying DPN or compression of the median and radial nerves. On the contrary, the opposite would be expected for the ulnar nerve, which would slacken in extension of the elbow. Further studies are necessary to evaluate the latter.

The first and major limitation of this study was that the ulnar nerve could not be analyzed with the applied methodology. This would have provided interesting information as the course ulnar nerve differs from the one of the median and radial nerve. The reason was that the nerve was found to be to slack and thin in all tested specimens to yield any significant tension. Further work is currently being undertaken to develop a biomechanical model using finer and fixed continuous nerve tension monitoring combined with a robotized arm to extend and further define the findings of this study. Secondly, since it is cadaveric, it does not take into account the effect of muscle tension and may not accurately reflect the in vivo situation. However, it may still provide valuable insights into the intraoperative setting since patients are often fully curarized during RSA implantation to facilitate exposure. Also, there is to this day no known threshold value in Human nerve strain beyond which clinical lesions may occur. Animal studies looking at lower-limb nerves have reported values as little as 6%-8% of strain beyond which clinical lesions could occur.^3^^,^^24^ Thirdly, due to the high amount of modularity in RSA and the high number of measurements required for this study, we only considered 2 implant configurations, using the smallest (P1) and largest (P2) configuration. We could not isolate the effect of distalization and lateralization.

## Conclusion

RSA significantly increases tension in the median and radial nerves and makes them more susceptible to wrist and elbow positioning. The mechanism behind DPN after RSA may thus result from increased compression of tensioned nerves against anatomical fulcrums rather than nerve elongation alone. Elbow flexion was the most effective way to decrease nerve tension, while elbow extension should be avoided when implanting the humeral component. Further studies are needed to assess the ulnar nerve.

## Disclaimers:

Funding: No funding was received for this work from any of the following organizations: 10.13039/100000002National Institutes of Health (NIH); Wellcome Trust; 10.13039/100000011Howard Hughes Medical Institute (HHMI); and other(s).

Conflicts of interest: The authors, their immediate families, and any research foundation with which they are affiliated have not received any financial payments or other benefits from any commercial entity related to the subject of this article.
